# Supplementary material for: Inhibitory Effects of Vandetanib on Catecholamine Synthesis in Rat Pheochromocytoma PC12 Cells
Source: Int J Mol Sci. 2025 Jul 18;26(14):6927. doi: 10.3390/ijms26146927 (PMC12295736; doi:10.3390/ijms26146927)
Supplement: Supplementary file 1 [file ijms-26-06927-s001.zip › ijms-3729535-Supplementary.pptx]

## Slide 1
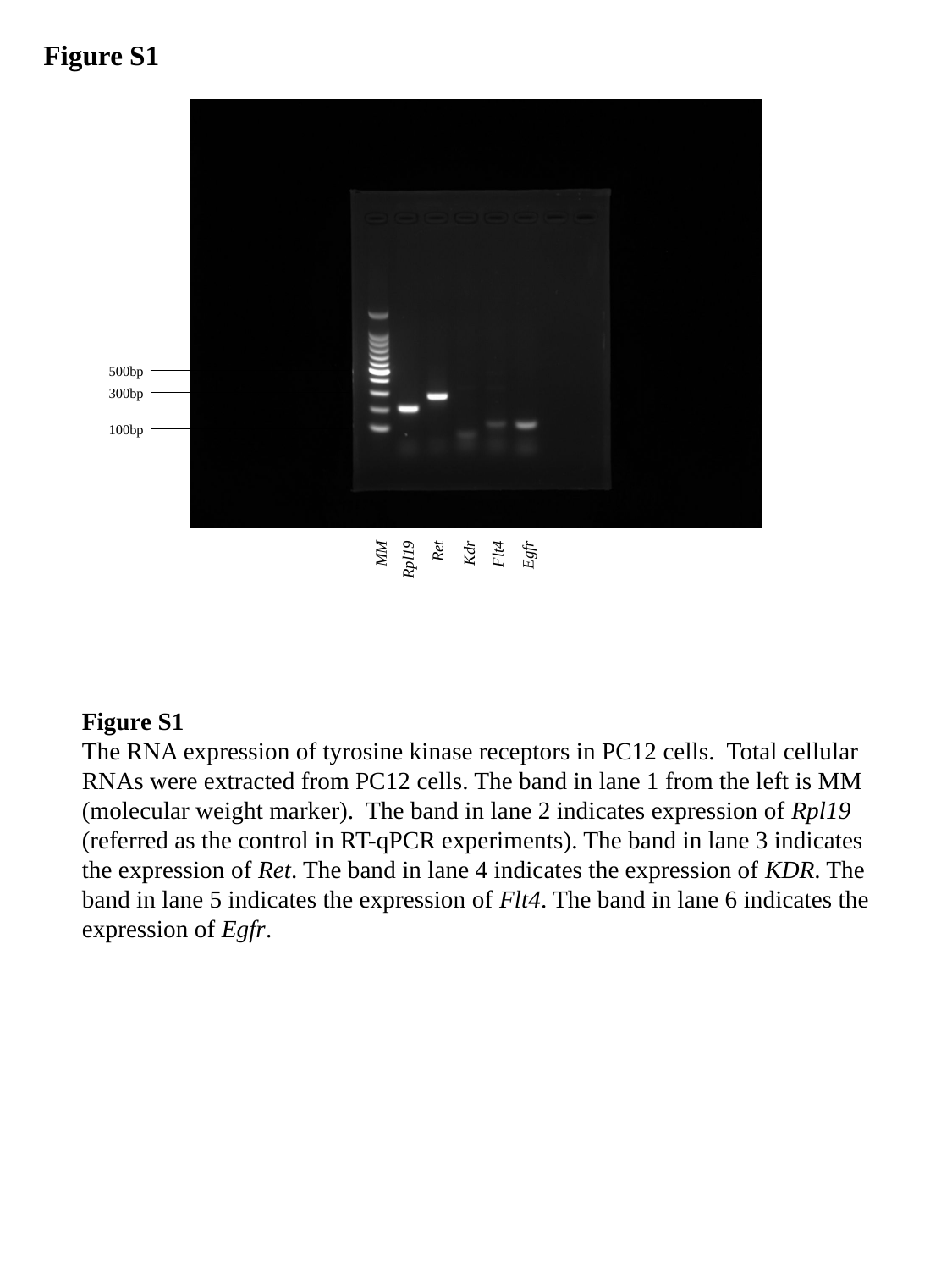

Figure S1
500bp
300bp
100bp
Kdr
Flt4
Egfr
Rpl19
　Ret
MM
Figure S1
The RNA expression of tyrosine kinase receptors in PC12 cells. Total cellular RNAs were extracted from PC12 cells. The band in lane 1 from the left is MM (molecular weight marker). The band in lane 2 indicates expression of Rpl19 (referred as the control in RT-qPCR experiments). The band in lane 3 indicates the expression of Ret. The band in lane 4 indicates the expression of KDR. The band in lane 5 indicates the expression of Flt4. The band in lane 6 indicates the expression of Egfr.

## Slide 2
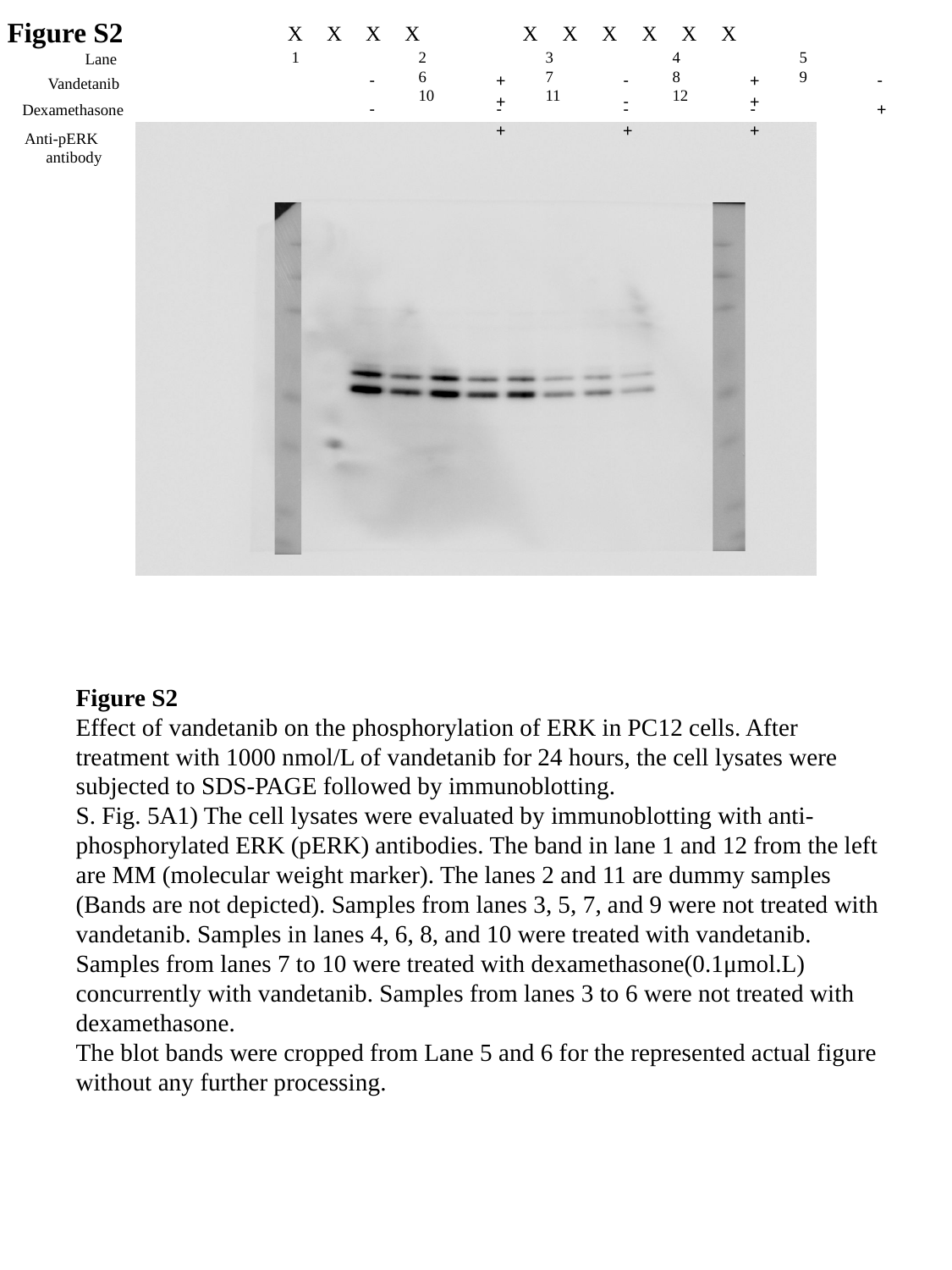

Figure S2
X
X
X
X
X
X
X
X
X
X
1	2	3	4	5	6	7	8	9	10	11	12
Lane
-	+	-	+	-	+	-	+
Vandetanib
-	-	-	-	+	+	+	+
Dexamethasone
Anti-pERK
antibody
Figure S2
Effect of vandetanib on the phosphorylation of ERK in PC12 cells. After treatment with 1000 nmol/L of vandetanib for 24 hours, the cell lysates were subjected to SDS-PAGE followed by immunoblotting.
S. Fig. 5A1) The cell lysates were evaluated by immunoblotting with anti-phosphorylated ERK (pERK) antibodies. The band in lane 1 and 12 from the left are MM (molecular weight marker). The lanes 2 and 11 are dummy samples (Bands are not depicted). Samples from lanes 3, 5, 7, and 9 were not treated with vandetanib. Samples in lanes 4, 6, 8, and 10 were treated with vandetanib. Samples from lanes 7 to 10 were treated with dexamethasone(0.1μmol.L) concurrently with vandetanib. Samples from lanes 3 to 6 were not treated with dexamethasone.
The blot bands were cropped from Lane 5 and 6 for the represented actual figure without any further processing.

## Slide 3
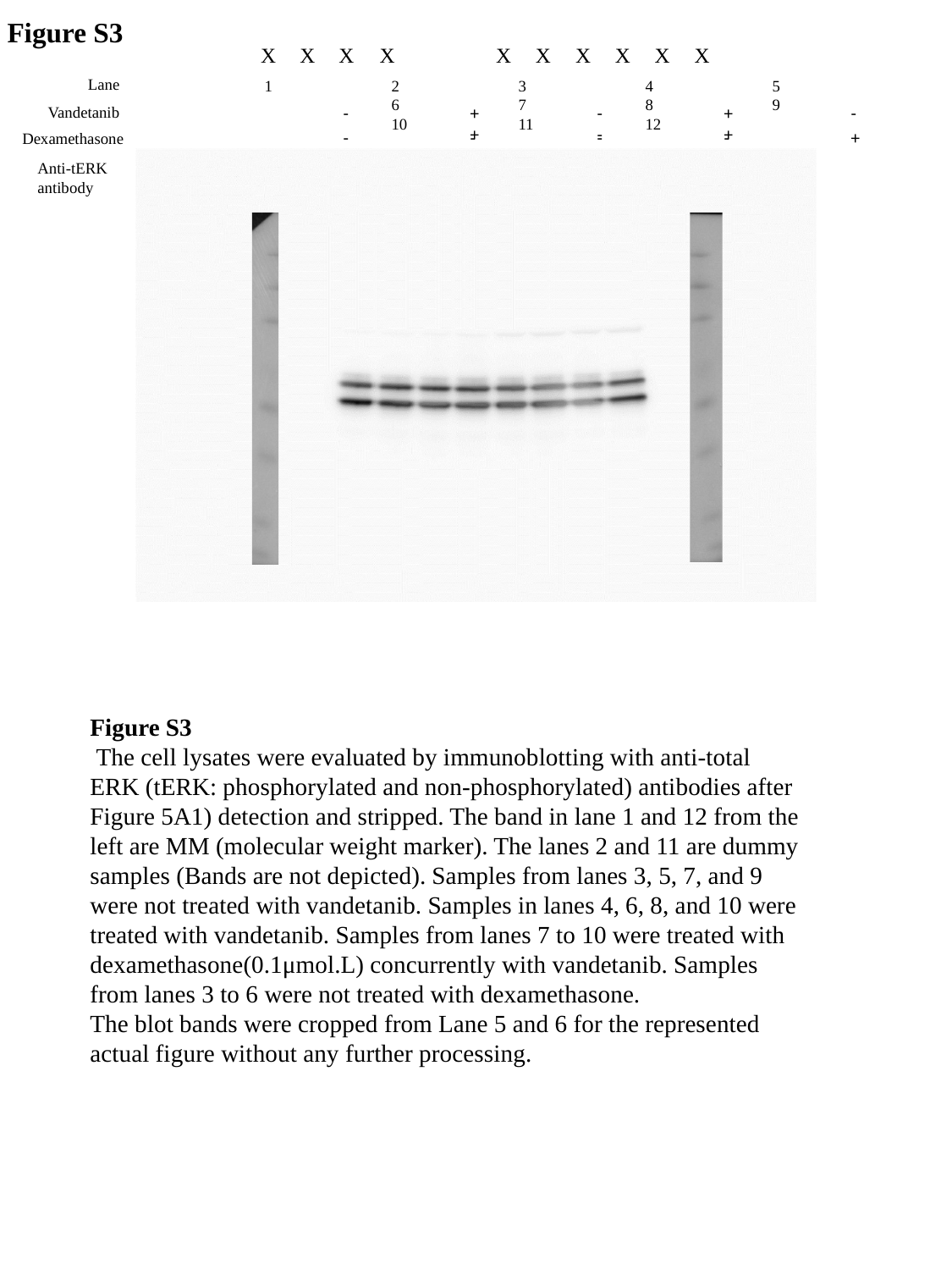

Figure S3
X
X
X
X
X
X
X
X
X
X
Lane
1	2	3	4	5	6	7	8	9	10	11	12
Vandetanib
-	+	-	+	-	+	-	+
-	-	-	-	+	+	+	+
Dexamethasone
Anti-tERK
antibody
Figure S3
 The cell lysates were evaluated by immunoblotting with anti-total ERK (tERK: phosphorylated and non-phosphorylated) antibodies after Figure 5A1) detection and stripped. The band in lane 1 and 12 from the left are MM (molecular weight marker). The lanes 2 and 11 are dummy samples (Bands are not depicted). Samples from lanes 3, 5, 7, and 9 were not treated with vandetanib. Samples in lanes 4, 6, 8, and 10 were treated with vandetanib. Samples from lanes 7 to 10 were treated with dexamethasone(0.1μmol.L) concurrently with vandetanib. Samples from lanes 3 to 6 were not treated with dexamethasone.
The blot bands were cropped from Lane 5 and 6 for the represented actual figure without any further processing.

## Slide 4
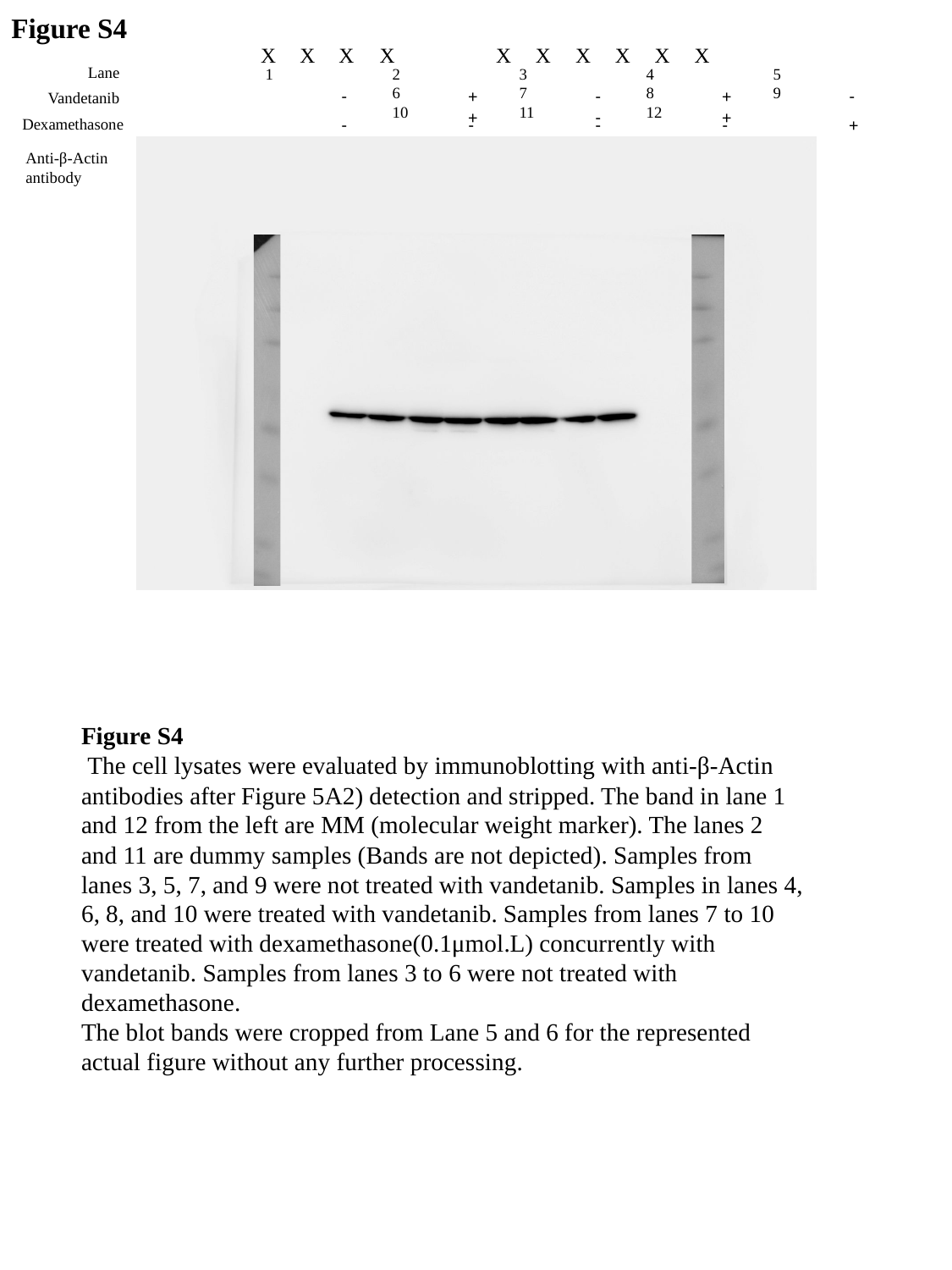

Figure S4
X
X
X
X
X
X
X
X
X
X
Lane
1	2	3	4	5	6	7	8	9	10	11	12
-	+	-	+	-	+	-	+
Vandetanib
-	-	-	-	+	+	+	+
Dexamethasone
Anti-β-Actin
antibody
Figure S4
 The cell lysates were evaluated by immunoblotting with anti-β-Actin antibodies after Figure 5A2) detection and stripped. The band in lane 1 and 12 from the left are MM (molecular weight marker). The lanes 2 and 11 are dummy samples (Bands are not depicted). Samples from lanes 3, 5, 7, and 9 were not treated with vandetanib. Samples in lanes 4, 6, 8, and 10 were treated with vandetanib. Samples from lanes 7 to 10 were treated with dexamethasone(0.1μmol.L) concurrently with vandetanib. Samples from lanes 3 to 6 were not treated with dexamethasone.
The blot bands were cropped from Lane 5 and 6 for the represented actual figure without any further processing.

## Slide 5
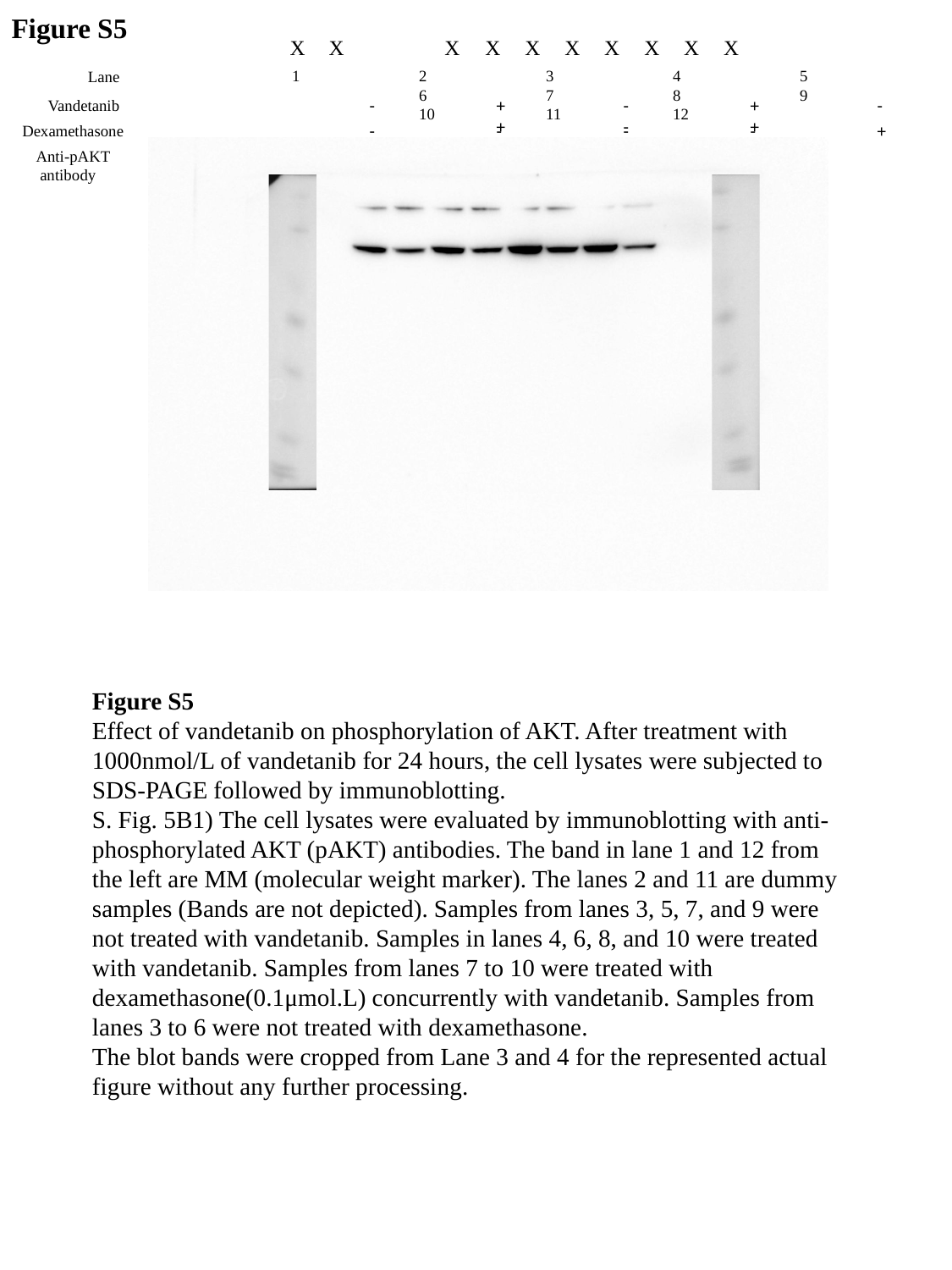

Figure S5
X
X
X
X
X
X
X
X
X
X
1	2	3	4	5	6	7	8	9	10	11	12
Lane
-	+	-	+	-	+	-	+
Vandetanib
-	-	-	-	+	+	+	+
Dexamethasone
Anti-pAKT
 antibody
Figure S5
Effect of vandetanib on phosphorylation of AKT. After treatment with 1000nmol/L of vandetanib for 24 hours, the cell lysates were subjected to SDS-PAGE followed by immunoblotting.
S. Fig. 5B1) The cell lysates were evaluated by immunoblotting with anti-phosphorylated AKT (pAKT) antibodies. The band in lane 1 and 12 from the left are MM (molecular weight marker). The lanes 2 and 11 are dummy samples (Bands are not depicted). Samples from lanes 3, 5, 7, and 9 were not treated with vandetanib. Samples in lanes 4, 6, 8, and 10 were treated with vandetanib. Samples from lanes 7 to 10 were treated with dexamethasone(0.1μmol.L) concurrently with vandetanib. Samples from lanes 3 to 6 were not treated with dexamethasone.
The blot bands were cropped from Lane 3 and 4 for the represented actual figure without any further processing.

## Slide 6
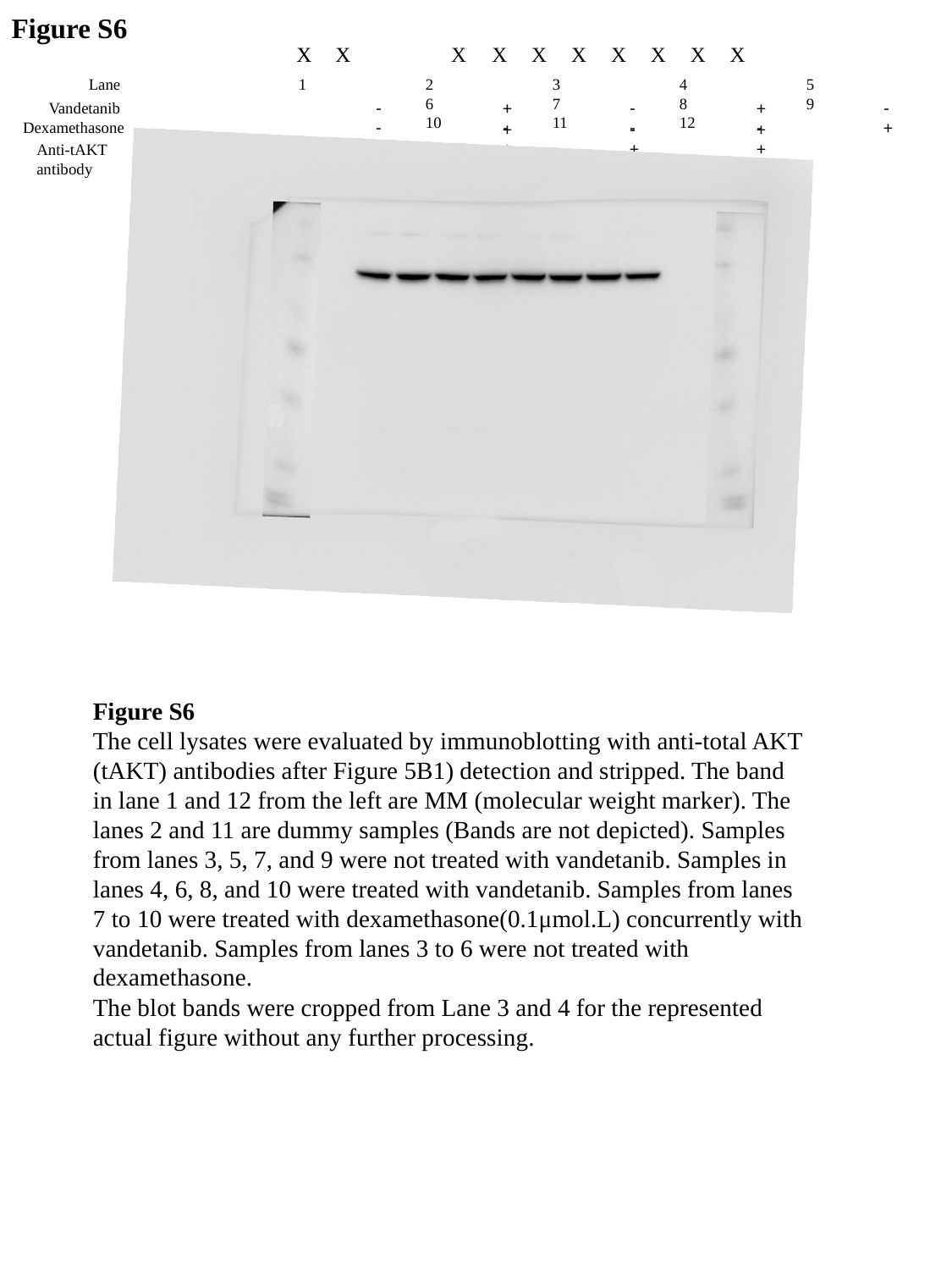

Figure S6
X
X
X
X
X
X
X
X
X
X
1	2	3	4	5	6	7	8	9	10	11	12
Lane
-	+	-	+	-	+	-	+
Vandetanib
-	-	-	-	+	+	+	+
Dexamethasone
Anti-tAKT
antibody
Figure S6
The cell lysates were evaluated by immunoblotting with anti-total AKT (tAKT) antibodies after Figure 5B1) detection and stripped. The band in lane 1 and 12 from the left are MM (molecular weight marker). The lanes 2 and 11 are dummy samples (Bands are not depicted). Samples from lanes 3, 5, 7, and 9 were not treated with vandetanib. Samples in lanes 4, 6, 8, and 10 were treated with vandetanib. Samples from lanes 7 to 10 were treated with dexamethasone(0.1μmol.L) concurrently with vandetanib. Samples from lanes 3 to 6 were not treated with dexamethasone.
The blot bands were cropped from Lane 3 and 4 for the represented actual figure without any further processing.

## Slide 7
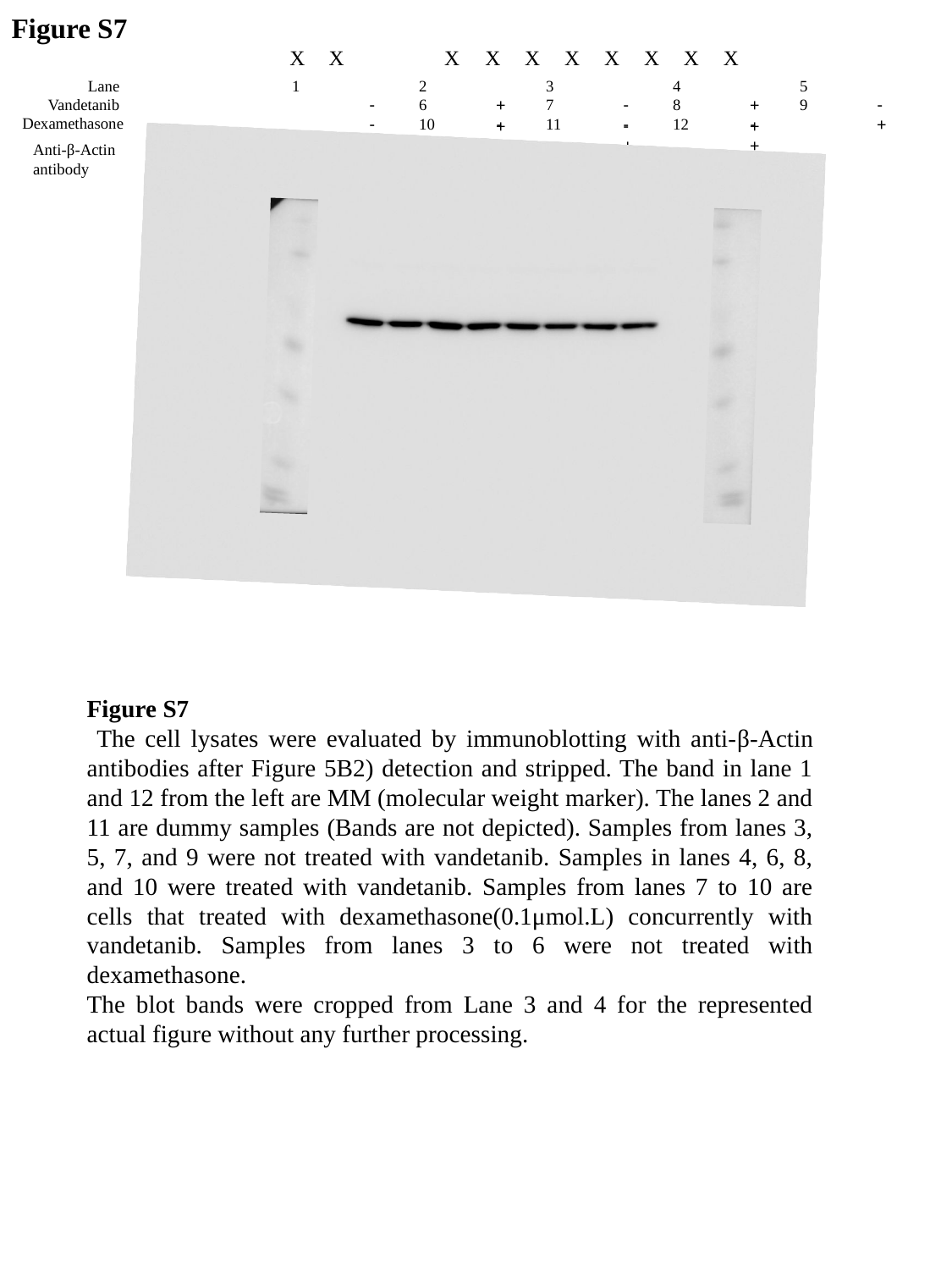

Figure S7
X
X
X
X
X
X
X
X
X
X
1	2	3	4	5	6	7	8	9	10	11	12
Lane
-	+	-	+	-	+	-	+
Vandetanib
-	-	-	-	+	+	+	+
Dexamethasone
Anti-β-Actin
antibody
Figure S7
 The cell lysates were evaluated by immunoblotting with anti-β-Actin antibodies after Figure 5B2) detection and stripped. The band in lane 1 and 12 from the left are MM (molecular weight marker). The lanes 2 and 11 are dummy samples (Bands are not depicted). Samples from lanes 3, 5, 7, and 9 were not treated with vandetanib. Samples in lanes 4, 6, 8, and 10 were treated with vandetanib. Samples from lanes 7 to 10 are cells that treated with dexamethasone(0.1μmol.L) concurrently with vandetanib. Samples from lanes 3 to 6 were not treated with dexamethasone.
The blot bands were cropped from Lane 3 and 4 for the represented actual figure without any further processing.

## Slide 8
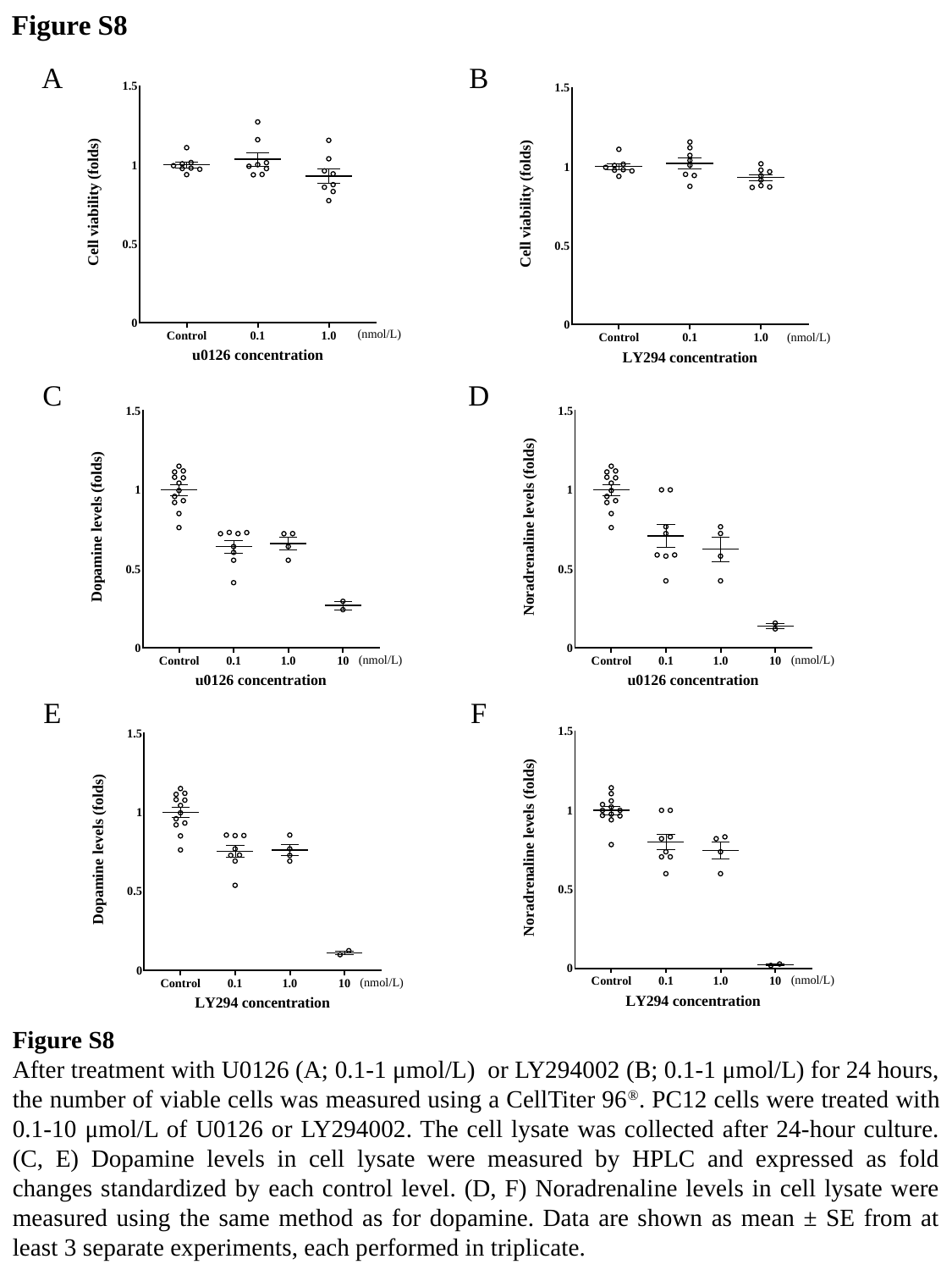

Figure S8
A
B
C
D
E
F
Figure S8
After treatment with U0126 (A; 0.1-1 μmol/L) or LY294002 (B; 0.1-1 μmol/L) for 24 hours, the number of viable cells was measured using a CellTiter 96®. PC12 cells were treated with 0.1-10 μmol/L of U0126 or LY294002. The cell lysate was collected after 24-hour culture. (C, E) Dopamine levels in cell lysate were measured by HPLC and expressed as fold changes standardized by each control level. (D, F) Noradrenaline levels in cell lysate were measured using the same method as for dopamine. Data are shown as mean ± SE from at least 3 separate experiments, each performed in triplicate.
